# The effect of extracellular vesicles on the regulation of mitochondria under hypoxia

**DOI:** 10.1038/s41419-021-03640-9

**Published:** 2021-04-06

**Authors:** Yaodan Zhang, Jin Tan, Yuyang Miao, Qiang Zhang

**Affiliations:** 1 Department of Geriatrics, Tianjin Medical University General Hospital, Tianjin Geriatrics Institute, Tianjin, China; 2 Tianjin Medical University, Tianjin, China

**Keywords:** Extracellular signalling molecules, Endosomes, Endosomes, Mitochondria

## Abstract

Mitochondria are indispensable organelles for maintaining cell energy metabolism, and also are necessary to retain cell biological function by transmitting information as signal organelles. Hypoxia, one of the important cellular stresses, can directly regulates mitochondrial metabolites and mitochondrial reactive oxygen species (mROS), which affects the nuclear gene expression through mitochondrial retrograde signal pathways, and also promotes the delivery of signal components into cytoplasm, causing cellular injury. In addition, mitochondria can also trigger adaptive mechanisms to maintain mitochondrial function in response to hypoxia. Extracellular vesicles (EVs), as a medium of information transmission between cells, can change the biological effects of receptor cells by the release of cargo, including nucleic acids, proteins, lipids, mitochondria, and their compositions. The secretion of EVs increases in cells under hypoxia, which indirectly changes the mitochondrial function through the uptake of contents by the receptor cells. In this review, we focus on the mitochondrial regulation indirectly through EVs under hypoxia, and the possible mechanisms that EVs cause the changes in mitochondrial function. Finally, we discuss the significance of this EV-mitochondria axis in hypoxic diseases.

## Facts

- Hypoxia directly regulates mitochondrial metabolism and mitochondrial compositions via HIF1α, and mitochondria can also trigger quality control mechanisms to adapt to hypoxia.
- As a medium of information transmission between cells, extracellular vesicles can be regulated by hypoxia.
- Extracellular vesicles containing mitochondria and mitochondrial DNA transferred between cells to induce inflammatory response of receptor cells.
- Extracellular vesicles can also regulate mitochondrial function through a variety of mechanisms.

## Open Questions

- Which specific substances contained in EVs plays a key role in the regulation of mitochondria?
- What are the specific mechanisms by which the contents of EVs enter directly into the mitochondria of recipient cells?
- Whether is there a mutual regulatory relationship among several pathways of mitochondrial transfer between cells, including EVs, tunneling nanotubes, exophers etc?

## Introduction

Mitochondria are important organelles for maintaining cell energy metabolism. However, researchers over the last few decades have shown that mitochondria are necessary to maintain cell biological function by transmitting information as signal organelles. mROS and the change of metabolites induced by hypoxic may cause the changes of nuclear gene expression, which is called mitochondrial retrograde signaling (MtRS) pathways. Mitochondria trigger multiple mechanisms to adapt to hypoxia, including the changes of oxidative phosphorylation, mitophagy, mitochondrial fission, and fusion^1^.

Hypoxia plays a crucial role in progression of disease as the main pathological mechanism of hypoxic diseases. In hypoxic diseases such as tumor^2^, ischemic disease^3^, mitochondria have multifunctional effects on cell damage and disease progression, and targeting mitochondria could be a therapeutic direction. For instance, it has been shown that metformin can inhibit cellular respiration and mitochondrial complex I activity of tumor cell to inhibit cancer progression^4^, and the combination of metformin and PD1 checkpoint blockers led to increased tumor-infiltrating T cells function and tumor clearance^5^. Thus, targeting mitochondria through drug intervention may change the disease process that are mediated by dysfunctional mitochondria.

In recent years, it has been found that EVs can be released by almost all types of cells. Hypoxia causes significant changes of the cargo, quantitative impact of secretion and the function of EVs. Mitochondria and their components can also spread between cells through EVs and other pathways, promoting inflammation, cancer progression, and the recovery of cellular function of damaged mitochondria. As a carrier of intercellular information transmission, EVs can carry active molecules and act on mitochondria of receptor cells, which cause the change of mitochondrial function. Thus, the change of mitochondria function and composition is not only the result of direct stimulation by hypoxia, but also likely the indirectly effect of EVs released by different physiological and pathological states of cells. We will review how EVs and their contents change mitochondrial composition, function, and possible mechanisms, which will provide ideas for the future application of EVs targeting mitochondrial pathway in hypoxic status.

## Extracellular vesicles, hypoxia and mitochondria

### Extracellular vesicles

EVs are membrane vesicles secreted by almost all cells, which can be divided into two main categories: exosomes and microvesicles, based on their biogenesis^6^. Microvesicles are formed by the outward budding of the plasma membrane, whereas exosomes are originated from intraluminal vesicles (ILVs), which formed by the inward budding of endosomal membrane in multivesicular endosomes (MVEs) and released by the fusion of MVEs and plasma membrane. Different EV subtypes have different sizes, density ranges, and contents, but they cannot be fully distinguished because of the overlap and technical limitations^7^. Although the physical characteristics, biogenesis and contents of different subpopulations of EVs are different, the biology of uptake by target cells are similar. Researchers have found that EVs play an increasingly important role in the intercellular communication by exchanging contents between cells^6,8–10^. In addition, each cell type may secrete different contents of EVs according to its physiological and stress state. Also, the production, secretion and uptake of EVs can be affected well, which results in the corresponding biological changes of receptor cells.

### Hypoxia

Hypoxia refers to oxygen limitation in tissue. In animals, oxygen can be exchanged in the lungs, and more than 95% of the oxygen will enter the capillaries through the exchange system of alveolar-capillary. Most tissues of the body obtain sufficient oxygen from capillaries to meet their basic metabolic requirements. Developing tumors rapidly outgrow their oxygen supply, the hypoxic tumor microenvironment ([PO_2_] < 10 mmHg, 1 mmHg O_2_ = 0.13% O_2_) is formed. It has been known that hypoxic condition promotes tumor progression, and it is necessary to improve hypoxic condition to treat tumor^11^.

Different tissues have different oxygen tension, with levels of large intestine as low as 0.5% O_2_ and up to 13% O_2_ in the lungs. However, the tissue oxygen levels of many major organs are 3–7%. Under the condition of mammalian cell culture, different degrees of the decline of cell oxygen concentration, such as hypoxia (0.5–2% O_2_) and severe O_2_ depletion (anoxic, <0.5% O_2_) have different degrees of cell damage^12,13^. In mild hypoxia (4% oxygen), non-cancerous macrophages migration is more related to HIF-1a-PDK1-mediated glycolytic reprogramming, and cytochrome C oxidase activity does not impair^14^, while the decline in mitochondrial oxygen availability may be the result of suppressing cytochrome C oxidase activity in severely hypoxic conditions. Different cellular oxygen concentrations regulate the glucose oxidation through different pathways, which may be the reason for different cellular stress responses. It is reported that moderate hypoxia (2.5% O_2_) in cultured spermatogonial stem cells can maintain mild ROS levels, which triggers the expression of the anti-apoptotic genes and promotes the proliferation^15^. Osrodek et al. showed that the phenotype of melanoma cells and their response to drugs were different in hyperoxia (21% O_2_), normoxia (6% O_2_) and hypoxia (1% O_2_), which might partly explain the differences between results in vitro and in vivo^16^. Thus, the definition of hypoxia needs to be further regulated for experiments in vitro.

### The role of mitochondria as signaling organelles

Mitochondria as the main oxygen-consuming organs are a cellular organelle with two membranes. The outer membrane encloses all the contents of the organelle, and the inner membrane and mitochondrial matrix contain many proteins, enzymes, and substrates involved in important metabolic reactions. Tricarboxylic acid (TCA) cycle generates the reducing nicotinamide adenine dinucleotide (NADH) and FADH2 via a series of metabolic reactions, which are required to produce ATP through the complexes of electron transport chain (ETC) in the inner mitochondrial membrane. The coupling mechanism between electron transport and ATP formation is known as oxidative phosphorylation (OXPHOS). Mitochondria are the main sites of ROS production, and mROS are generated primarily by complexes I^17^ and III^18^ of the ETC, and complex II may also serve as a contributor of ROS under hypoxia^19^. The balance of mitochondrial fusion and fission regulates mitochondrial morphology network, which is known as mitochondrial dynamics. Mitochondrial fission is regulated by Dynamin-related protein 1 (Drp1) and Dynamin2 (Dnm2), and mitochondrial fusion can be divided into outer membrane fusion and inner membrane fusion, which are mediated by mitofusins1 and 2 (Mfn1 and Mfn2) and opticatrophy1 (OPA1), respectively^20^. Damaged mitochondria induce mitochondrial fission under stress, which promote mitophagy and allow them to be cleared by mitophagy.

**Fig. 1:**
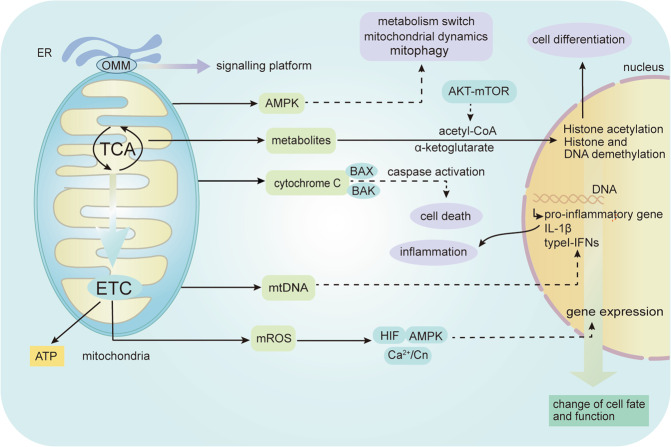
The role of mitochondria as signaling organelles. Mitochondria play greatly important roles in generating ATP through oxidative phosphorylation (OXPHOS). The main mitochondrial-dependent signaling events: the release of mitochondrial ROS to induce the changes of gene expression through HIF/AMPK/Ca^2+^/Cn; release of metabolites to induce histone acetylation, histone and DNA demethylation, which determined cell differentiation; activation of AMPK regulates mitochondrial metabolism, mitophagy, and mitochondrial fission and fusion; release of cytochrome C activates caspases cascade reaction to induce cell death; release of mtDNA causes the secretion of pro-inflammatory cytokines. Moreover, mitochondrial outer membrane (MOM) can be used as a signaling platform. B cell lymphoma-2 (BCL-2)-associated X protein (BAX) and BCL-2 antagonist/killer 1 (BAK), endoplasmic reticulum (ER), Ca2+/ Calcineurin (Cn), hypoxia inducible factor (HIF), Adenosine monophosphate‑activated protein kinase (AMPK).

Mitochondria can support cell reproduction and growth through their role of biosynthesis and bioenergy. However, it has been found over the past two decades that mitochondria play an increasingly important role in determining gene expression profile and controlling signal pathways. In the past, eukaryotic mitochondria, regulated the adaptive response of cells by the production of signal substances such as acetate and ROS, which promote acetylation by acetyl-coA and oxidation of proteins, respectively^21^. Subsequently, it has been found that mitochondria release more signal components into the cytoplasm to regulate biological functions by affecting the other parts of the cells under hypoxia, including the release of ROS, metabolites, cytochrome C, mitochondrial DNA (mtDNA), and activation of AMPK. Moreover, mitochondrial outer membrane can be used as a signaling platform (Fig. 1).

Mitochondrial-mediated cell death pathway plays an important role in regulation of cardiomyocyte death^22^. The main event of mitochondrial apoptosis pathway is the increase of mitochondrial outer membrane permeability, which leads to the release of mitochondrial cytochrome C to activate caspases cascade reaction to induce cell death^22^. Covarrubias et al. demonstrated that Akt-mTORC1 pathway control the production of acetyl-CoA by regulating ATP citrate lyase, which affect the histone acetylation and determine macrophage activation^23^. In addition, metabolites such as succinate, fumarate, α-ketoglutarate, L-2-HG regulate histone, and DNA demethylation^24^. Cell stress cause mitochondrial damage and the release of mtDNA, which enhance pro-inflammatory responses through TLR9 pathway or inflammasome activation^25^. In addition, mtDNA can increase expression of type I IFNs through multiple pathways and mechanisms, which has been comprehensively described elsewhere^25^. Under mild hypoxia, low level production of mROS is important for maintaining normal mitochondrial function^26,27^. Also, mROS can induce multiple pathways of MtRS under hypoxic conditions, including the Ca^2+^/ Calcineurin (Cn) pathway, the HIF pathway, and the AMPK pathway. Chowdhury et al. demonstrated that Ca^2+^/Cn pathway is more sensitive to mROS, partly because of the destruction of mitochondrial membrane potential^28^. AMPK can maintain mitochondrial homeostasis, including the switch of mitochondrial anabolism and catabolism, the regulation of mitochondrial fission/fusion and mitophagy^29^.

## Mitochondria and EVs under hypoxia

### The changes of mitochondria under hypoxia

Hypoxia as a major stressor, has been seen the significant feature of pathological states, such as stroke, tumor, ischemic heart disease, inflammation, obesity, or obstructive sleep apnea. Hypoxia plays a vital role in cell differentiation, inflammation, death by regulating the composition and function of mitochondria^1,30,31^. What’s more, mitochondria can also evoke adaptive mechanisms to maintain mitochondrial function in response to hypoxia stress.

The TCA cycle can be inhibited under hypoxia, limiting the generation of reducing equivalents, diminishing electron flux through the ETC, which also affects sugar and lipid metabolic pathways^13^. Prolylhydroxylases (PHD) is a receptor and adapter for hypoxia. The loss of PHD activity under hypoxia stabilizes the HIFα. HIF-1α promotes glycolytic reprogramming by inducing pyruvate dehydrogenase kinase, isozyme 1 (PDK1), which inhibits pyruvate dehydrogenase (PDH) to prevent conversion of pyruvate to acetyl-CoA, reducing the TCA cycle flux in mild hypoxia^14^.

Also, sustained hypoxia can decrease ETC activity, although the inhibitory effect of acute hypoxia on ETC may not be obvious. The activity of ETC began to decrease when the intracellular oxygen concentration was 0.3%^32^, which may be related to the high affinity of cytochrome C oxidase (also known as complex IV or COX) for oxygen, so that ETC could maintain ATP levels under anoxia. Fukuda et al. found that hypoxia regulates the switch of COX4 subunits by HIF-1 activation for a more efficient transfer of electrons to O_2_ to meet energy demands in mammals^33^.

At the same time, hypoxia-induced moderate mROS has various signaling roles, and mROS needs to be tightly regulated to avoid its overproduction. Cells exposed to hypoxia for a long time will decrease the activity of ETC to limit the production of mROS^34^. However, the inhibition of mROS is limited.

**Fig. 2:**
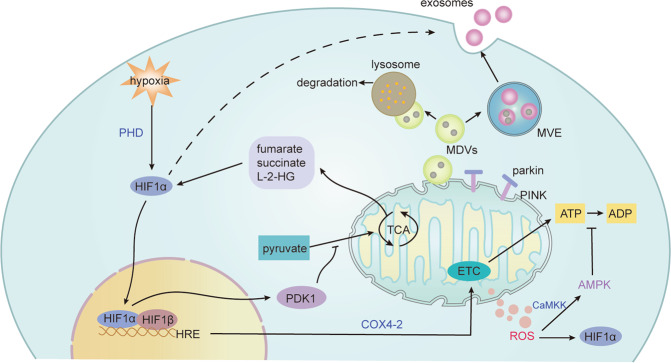
Mitochondrial changes under hypoxia. The tricarboxylic acid (TCA) cycle is inhibited under hypoxia, and HIF-1α prevents pyruvate from entering the TCA cycle by inducing pyruvate dehydrogenase kinase, isozyme 1 (PDK1). Hypoxia also regulates the activity of the electron transport chain (ETC). The switch of cytochrome C oxidase4 (COX4) subunits by HIF-1 activation for a more efficient transfer of electrons to O_2_ to meet energy demands in mammals under acute hypoxia, but sustained hypoxia can decrease ETC activity. The decreased activity of ETC limit the production of mROS, which mediates signal transduction and oxidative damage. The increase of mROS activates CaMKK, which activates AMPK to limit ATP consumption. Also, TCA cycle metabolites fumarate, succinate, L-2-hydroxyglutarate (L-2-HG), as well as mROS can cause the accumulation of HIFα. In addition, mitochondrial fission and fusion and mitophagy also play an important role in adaptation to hypoxia. Also, EVs and mitochondrial-derived vesicles (MDVs) can be effective mechanisms to maintain mitochondrial quality control. Damaged mitochondria and compositions can be removed by EVs. Also, mitochondrial stress induces the formation of MDVs partly depending on parkin and PINK1, which can fuse with multivesicular endosomes (MVEs) or lysosomes, and the contents can be secreted to the extracellular space via exosomes or degraded, respectively. Ca^2+^/calmodulin-dependent protein kinase kinase (CaMKK).

Mitophagy and mitochondrial fission and fusion also play an important role in adaptation to hypoxia^35^. Hypoxic-induced mROS led to the increase in mitochondrial fission, which triggers cisplatin resistance as a process of cellular adaption to the hypoxic tumor microenvironment^36^. Detached cancer cells gather together to cause the hypoxic environment that promotes HIF1α mediated mitophagy, removing damaged mitochondria, supporting glycolysis, which promotes cancer cell progression. Disruption of metabolic adaptation or cell clusters leads to a decline of the metastatic ability of tumor in vivo^37^. The regulation of mitochondria in tumor cells makes them more tolerant to the hypoxia environment and promotes tumor progression^38^, but targeting mitochondrial function for the therapeutic potential is still controversial because of the non-specifically suppression of anticancer immune responses. Thus, mitochondrial specific targeting therapy still needs a lot of work. In addition, EVs and mitochondrial-derived vesicles (MDVs)^39–41^ can be effective mechanisms to preserve mitochondrial homeostasis, which will be discussed below (Fig. 2).

### The change of EVs under hypoxia

Hypoxia affects not only the amount of secreted EVs, but also the contents and corresponding functions. Hypoxic melanoma cells released more EVs than normoxic cells, and hypoxic melanoma EVs showed specific proteins and miRNA characteristics, which might be closely related to the poor prognosis of melanoma patients and might be a potential biomarker for melanoma^42^. Hypoxia tumor cells derived exosomes contain more chemokines, immunosuppressive proteins, and miRNA, which promote tumor progression by regulating phenotypic differentiation of macrophages^43,44^. Researchers have summarized the molecular mechanisms participated in hypoxia-induced exosomes biogenesis and cargo loading (miRNAs, proteins, and lipids) in cancer cells^45,46^, including HIF-1α, ceramides, RAB GTPases, ROCK, tetraspanins, oxidative stress. Furthermore, the change of exosomal cargo under hypoxia promotes the communication between tumor cells and stroma^46^, which involved in tumor angiogenesis, survival, proliferation, immunomodulation, invasion, metastasis, and drug resistance^47,48^. What’s more, the production of EVs under hypoxia also occurs in non-cancerous cells, and HIF1α are key regulators of EVs production under hypoxia^49,50^. For instance, the release of hypoxic tubular epithelial cells derived miRNA-23a-enriched exosomes is dependent on HIF1α^51^. At the same time, the release of exosomes enriched with miR-216a-5p derived from mesenchymal stem cells(MSCs) under hypoxia was significantly higher than the normoxic conditions, and was also more easily taken up by microglia^52^. Bronchoalveolar lavage fluid derived miRNA-enriched-EVs may be more likely endocytosed by alveolar macrophages to promote inflammatory response^53^. Therefore, the changes of cargo contained in EVs under hypoxia affect their uptake by target cells. Exosomal miRNA also regulates tissue response to hypoxia^54^. In addition, oxygen concentration can also determine EV release. Exosomes isolated from human cardiac progenitor cells had a significantly increase under physoxia conditions (5% O_2_) compared with normoxia (21% O_2_) and hypoxia (1% O_2_)^55^.

### Biology of EVs uptake

**Fig. 3:**
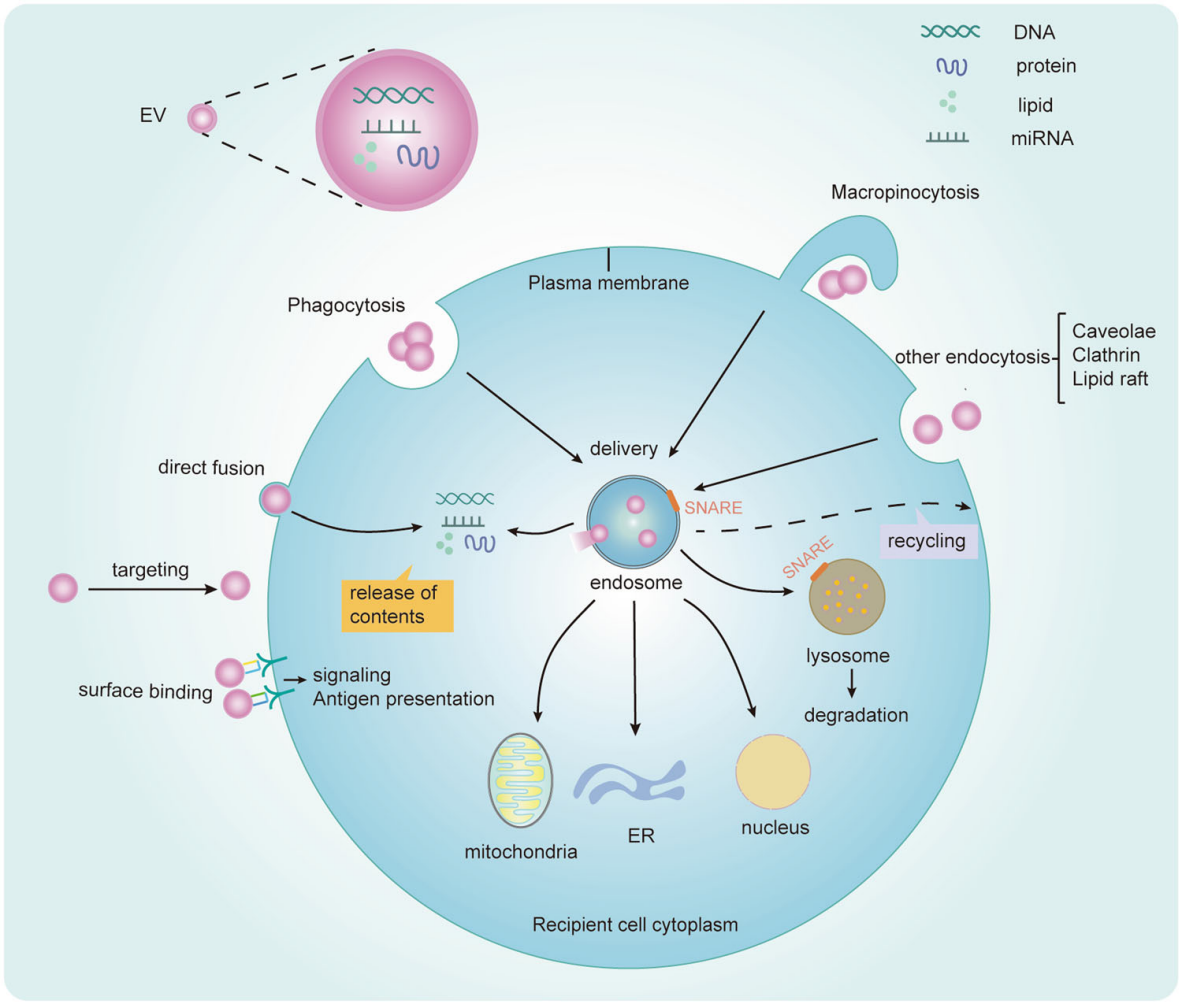
Biology of EVs uptake by recipient cells. (1) EVs mediated intercellular communication requires targeting the receptor cells. (2) In the recipient cell, EVs will bind to the cell surface and initiate intracellular signaling pathways through receptor ligand binding. EVs may also be internalized by acceptor cells through multiple pathways, including macropinocytosis, phagocytosis, clathrin-dependent endocytosis, caveolin-mediated uptake, and endocytosis via lipid rafts, as well as fuse with the plasma membrane of recipient cells. (3) EVs can release contents to the cytoplasm of the recipient cell by fusion with the plasma membrane and endosome membrane, and also direct transfer into the nucleus, endoplasmic reticulum or mitochondria through the contact of these organelles with endosomes that contain internalized EVs. The most important outcome of EVs is targeted to lysosomal for degradation and also be possibly recycled. SNARE proteins are required for intracellular vesicles fusion.

Hypoxia promotes the release of EVs into the extracellular environment, which can be taken up by cells at the distant or nearby. EVs induces functional responses of recipient cells by transferring their contents. The uptake of EVs by receptor cells requires three steps (Fig. 3).

The first step of EVs uptake is targeting receptor cells, which may be determined by the source or surface components of EVs, or even the contents of EVs, as well as receptors on the surface of receptor cells. Interacting molecules between EVs and receptor cells are known, including integrins, lipids, tetraspanins, intercellular adhesion molecules etc, which have been systematically reviewed^6^. For example, integrins determine the specificity of tumor cell exosomes targeting metastatic organs^56^. However, exogenous EVs usually lack targeting specificity. Some studies have shown that exogenous EVs enter the body and mainly gather in the liver, lungs, and spleen. Other studies have shown that the targeting specificity of EVs can be increased by modifying EVs. For example, bone marrow MSCs derived exosomes can enhance the specificity and efficiency of targeting ischemic myocardium by engineering ischemic myocardium-targeting peptide, and enhance the therapeutic effect on acute myocardial infarction^57^. Yerneni et al. demonstrated that uptake and targeting specificity of EVs can be specifically altered with a tethered protein via membrane tethering of oligonucleotides, such as functionalizing exosomes with an immunomodulatory protein-FasL^58^. Luo et al. generated a endogenous exosome tracking mouse model to satisfy various research goals^59^, and Verweij et al. developed an zebrafish embryos model to study the biogenesis, composition, journey, and fate of EVs^60^. They found that the large number of yolk syncytial layer exosomes are endocytosed by endothelial cells and macrophages by a combination of imaging methods and proteomic analysis, which highlight the targeting specificity of exosomes^60^. However, the molecular basis for determining the targeting specificity of EVs still needs to be further explored.

The next step of uptake is the combination of EVs with recipient cells. EVs can transmit signals to receptor cells through surface binding, but not deliver contents. EVs isolated from dendritic cells have been shown to activate T lymphocyte receptors^61^ or present antigens to T cells via surface contact^62^. Many studies have shown that EVs can be internalized by receptor cells, including macropinocytosis, phagocytosis, clathrin-dependent pathways, and caveolin-mediated endocytosis, as well as through endocytosis via lipid rafts^6,63,64^.

The final step is the delivery of contents to the recipient cells and intracellular fate of EVs. The contents of EVs are released to the recipient cells by direct fusion with the plasma membrane^65,66^. Importantly, the endosomal pathway is also the main pathway for the release of contents^7^, including EVs-endosome back fusion^67–69^, the contact of endosomes that contain internalized vesicles with organelles such as nucleus^70^, the endoplasmic reticulum^71,72^ or mitochondria^72^. Perhaps the most vital fate of EVs is to target lysosome for degradation, and also possibly be recycled to the plasma membrane. The Placental syncytiotrophoblast EVs were internalized into endosomes and delivered specific miRNAs to the mitochondria or endoplasmic reticulum of recipient cells depending on the state of the source cells of EVs^72^. Chen et al. demonstrated that anti-angiogenic proteins entered the endosome via endocytosis, and transferred to mitochondria through membrane fusion or kiss-and-run mechanism between late endosome and mitochondria using confocal and super-resolution fluorescent microscopy^73^. However, the detailed mechanism and the corresponding influencing factors of transmitting EVs contents from endosomes to organelles are still unclear, which is of great significance for in-depth understanding of the intracellular fate and function of EVs.

### The transfer of mitochondria between cells

**Fig. 4:**
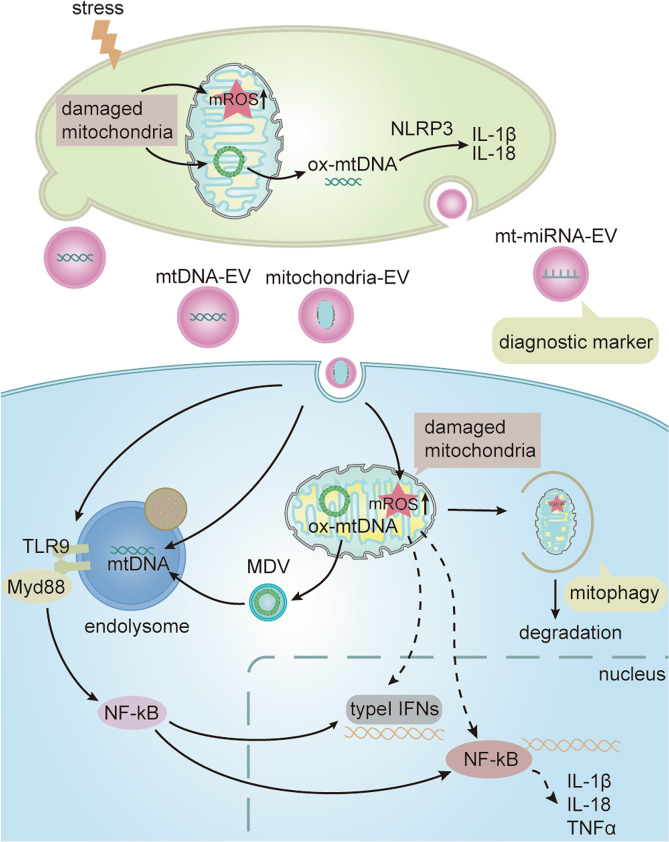
The role of mitochondria and mitochondrial compositions transferred between cells via EVs. Damaged mitochondria lead to the release of mROS, oxidative mtDNA and mitochondria or its components-containing EVs under stress(hypoxia). (1) The oxidative mtDNA in resident cells increases inflammatory cytokines such as IL-1β and IL-18 through activating NOD (Nucleotide binding oligomerization domain)-like receptors (NLRP3) inflammasome pathway. (2) mtDNA that enter endolysome pathway through endocytosis into the receptor cells via EVs and by mitochondria-derived vesicles (MDVs) acts on Toll-like receptor 9 (TLR9)–myeloid differentiation primary response protein 88 (MYD88)–nuclear factor-kB (NF-kB) signaling for pro-inflammatory gene expression. (3) Damaged mitochondria enter into the recipient cells via EVs, which induce the generation of mROS that lead to the increased secretion of inflammatory cytokines, such as tumor necrosis factor (TNF) and Type I interferon (IFN) in recipient cells. (4) In addition, damaged mitochondria can be degraded through mitophagy, and mitochondrial miRNA and protein can be detected as diagnostic markers.

It has been found that some cells can secrete EVs containing mitochondria or mitochondrial compositions, such as monocytes^9^, hepatocytes^74^, endothelial cells^75^, and MSCs^76,77^, and their effect on recipient cells is determined by the activated status of their source of cells. For instance, damaged mitochondria and composition can be removed by EVs^40,76^. Researchers believed that the transfer of functional mitochondria or mitochondrial compositions via EVs may increase cellular bioenergetics in ischemic endothelial cells with decreased energy levels to be strategies for treating stroke^78^. Also, EVs derived from MSCs^76,77^and renal scattered tubular cells^79^ have positive effects on recipient cells by transmitting mitochondria. But not all MSCs-derived EVs contain mitochondria. Thomas et al. showed that 25% of MSCs-EVs were positive for mitochondria^10^. In addition, the increased release of EVs containing mitochondrial miR-494-3p^80^ and membrane proteins^81^ may represent a diagnostic marker for mitochondrial injury. Also, mitochondrial dysfunction in astrocytes can be reflected by depletion of mitochondrial components in EVs, which may also be a diagnostic method^82^. What’s more, lipopolysaccharides activated monocytic cells results in the release of free mitochondria, as well as microvesicles enriched in mitochondria, which induce TNF-α and type I interferon-(IFN) signaling pathway, leading to the proinflammatory effects on receptor cells^9^. Moreover, the transfer of mitochondria via exosomes derived from pro-inflammatory myeloid-derived regulatory cells results in its co-localization with the mitochondrial network of the T cells to produce ROS^83^. Endothelial microparticles (EMPs) derived from lipopolysaccharide-treated endothelial cells contain dysfunctional mitochondria, which contributed to the EMPs-mediated inflammatory response^75^. Mitochondrial Lon induced the release of EVs-containing mtDNA, which enhances the M2 macrophages function through the TLR9-dependent pathway and inhibits CD8+ T cell activity, promoting tumor progression^84^. Tsilioni et al. found that EVs-containing mtDNA derived from the serum of patients with autism spectrum disorder promote the secretion of more pro-inflammatory factor IL-1β in human microglia^85^ (Fig. 4).

Also, mitochondrial stress induces the formation of MDVs with sizes from 70 to 150 nm, which have been considered as a pathway of mitochondrial quality control by transferring specific mitochondrial contents into endolysosomal system^41,86–88^. In endosomal–lysosomal system, MDVs can fuse with MVEs or lysosomes, and the mitochondrial contents can be secreted to the extracellular space via exosomes or degraded, respectively. The release of MDVs increased under neuronal stress^89^, but the functional significance is still few studied. The pathogenic bacteria induces the production of mROS to promote the bactericidal ability of macrophages, which is delivered to phagosomes by MDVs^90^. Also, MDVs also involve the mitochondrial antigen presentation to trigger the immune response^91^. Therefore, it is reasonable to consider that oxidized mtDNA enters into the endolysomal pathway through the internalization of EVs or by MDVs, which activates TLR9 Inflammatory pathways and increases the pro-inflammatory gene expression (Fig. 4). Interestingly, exogenous MDVs under different hypoxic conditions mitigated hypoxia-induced myocardial injury and mitochondrial apoptosis via conveying Bcl-2 in vitro^92^. In brief, the contents of MDVs determine the biological effects, depending on the conditions of the cells^93^. A novel high-resolution density gradient^93^ is necessary for the further development of EVs and MDVs-related research.

It’s interesting that there are many different ways for the transfer of mitochondria between cells, including tunneling nanotubes (TNTs), exophers^94,95^, cellular fusion, and GAP junctions^95^. TNTs are membranous channels for cell communication, allowing transport of intercellular signaling and organelles between heterogeneous cells. Many studies have shown that mitochondrial transfer between tumor cells as a mechanism of metabolic adaptation promotes tumorigenesis, invasiveness and metastasis^96–98^. And preventing the formation of TNTs may be a novel approach to treat cancer^99,100^. Researchers showed that TNTs formation and the transfer of cargo between neuronal cells are regulated by the Wnt/Ca^2+^ pathway, thus intervention of Wnt/Ca^2+^ pathway may be a way to reduce TNTs-caused damage^101^. However, renal CD133+ scattered tubular cells transfer mitochondria through TNTs to injured tubular cells under hypoxia, which plays a cellular protective effect to restore the function of damaged mitochondria^102^. In general, mitochondrial damage may be an important trigger for mitochondrial transfer through TNTs.

Recently, Hayakawa et al. found that stoke induces mitochondria of astrocyte entry to neighboring neurons through extracellular mitochondria particles with the range of sizes from 300 to 1100 nm, which promotes neuronal viability^94^. Lately, some researchers found that exophers (average size 3.8 μM) containing damaged mitochondria extruded by neurons^103^ or cardiomyocyte^104^ were secreted to extracellular space or cleared by macrophages, which can improve mitochondrial quality control and homeostasis, maintaining the function of neurons and cardiomyocytes, respectively. Thus, the intercellular transfer of mitochondria can be used as an adaptive mechanism to maintain mitochondrial health and improve cellular energetics of hypoxic cells, which may be an effective therapeutic strategy.

## The regulation of mitochondria by EVs

### Mitochondrial regulation by EVs and their contents

**Fig. 5:**
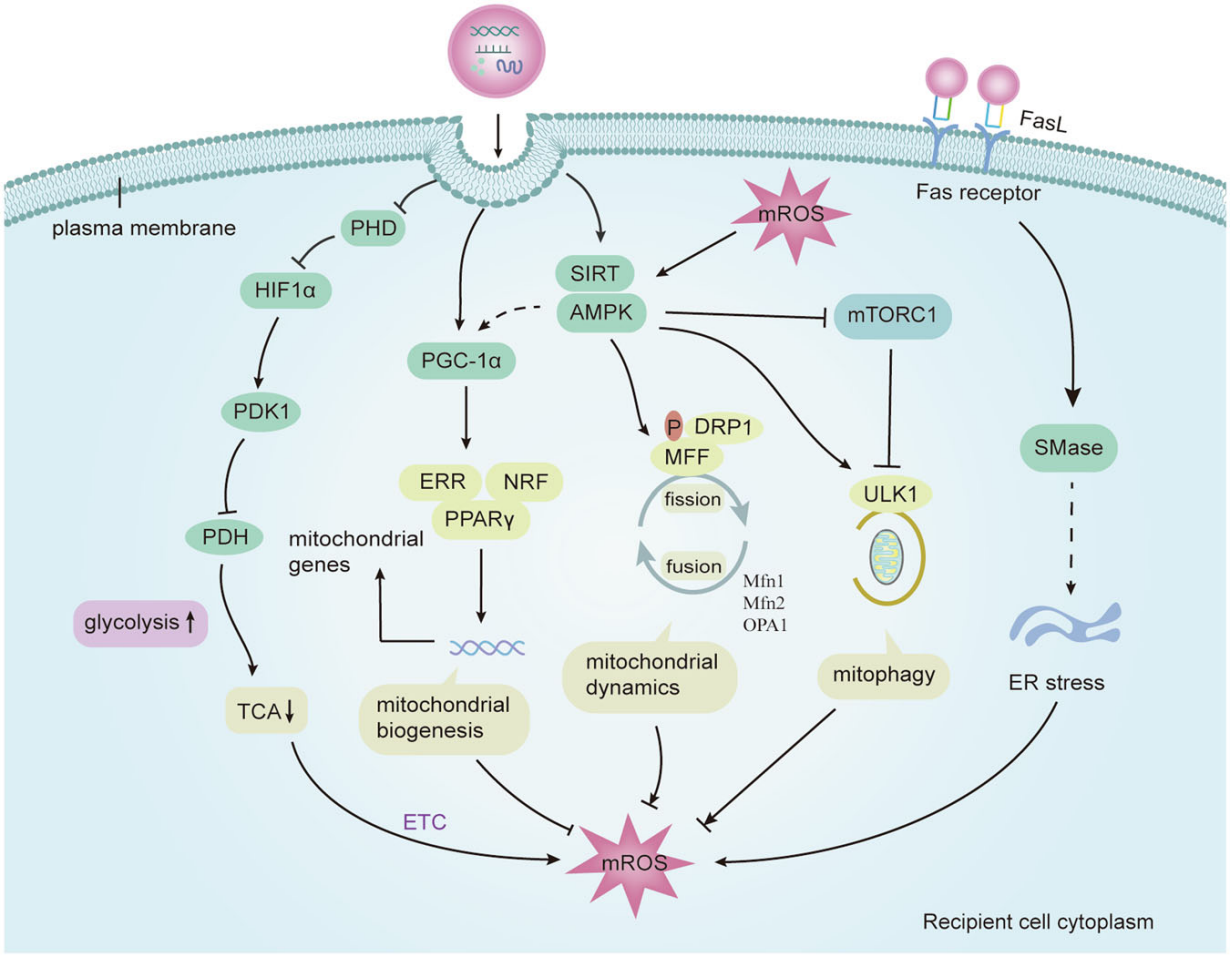
The mechanism of mitochondrial regulation by EVs. EVs cause changes in the mitochondrial function of the receptor cells by releasing the contents to the target cells, thus changing the fate of the cells. (1) EVs and their active molecules enhance the glycolysis of recipient cells by binding PHD2 and preventing the degradation of HIF-1α, which inhibits TCA cycle by inducing PDK1 that inhibits pyruvate dehydrogenase (PDH) activity; (2) EVs activate PGC-1α by directly targeting or through AMPK, which promotes mitochondrial biogenesis by interacting with PPARγ, ERRs, or NRF; (3) EVs activate AMPK, which regulates mitophagy and mitochondrial fission by activating ULK1 and directly phosphorylates MFF through DRP1, respectively; (4) EVs affect endoplasmic reticulum stress through Fas ligand receptor pathway, which promotes the production of mitochondrial ROS and ultimately affects mitochondrial function. In addition, mROS is regulated by mitochondrial metabolism, mitochondrial biogenesis, mitochondrial fission and fusion and mitophagy, and mROS can also regulate AMPK activation. MFN1/2 mitofusin 1/2, OPA1 optic atrophy 1, DRP1 dynamin-related protein 1, MFF mitochondrial fission factor, ULK1 unc-51 like autophagy activating kinase 1, NRF nuclear respiratory factors, PPARγ proliferator-activated receptor-γ co-activator 1α (PGC1α), ERRs estrogen-related receptors, TCA tricarboxylic acid, ETC electron transport chain, HIF hypoxia inducible factor, AMPK adenosine monophosphate‑activated protein kinase, SIRT sirtuin, mROS mitochondrial reactive oxygen species, mTOR mammalian target of rapamycin, mTORC1 complex 1, PHD prolylhydroxylases, pyruvate dehydrogenase kinase1 (PDK1), PDH pyruvate dehydrogenase.

Some studies have shown that changes in mitochondrial function can affect the production and release of EVs^105–107^. For instance, laminar shear stress caused by aerobic exercise increases the biogenesis of mitochondria via the mechanism of SIRT1/PGC1α dependence, which reduces the production of EMPs^105^. Iron overload induces mitochondrial damage and apoptosis of endothelial cells, resulting in increased release of EMPs, which can be reduced by decreasing endothelial mitochondrial injury and ROS generation treated with deferiprone^106^. Moreover, researchers found that mitochondria function and composition were indirectly affected by the release of EVs, except for the direct regulations under hypoxia. EVs carry a variety of metabolites, proteins, and miRNA to regulate mitochondria of receptor cells, and affect cell differentiation and disease progression. Zhao et al. demonstrated that cancer-related fibroblasts deliver many metabolites to cancer cells via exosomes to provide substances for TCA cycle, promoting cancer progression^108^. Also, adipocyte EVs deliver proteins and fatty acid substrates to stimulate fatty acid oxidation in melanoma cells, modifying the mitochondrial dynamics, which promotes melanoma migration^109^. In brief, EVs can regulate mitochondrial metabolism, biogenesis, mROS, mitophagy and mitochondrial fission and fusion, and the relevant mechanisms will be discussed in detail next (Fig. 5).

### The mechanism of mitochondrial regulation by EVs and their contents

#### HIF1α/PDK1 pathway

HIF1α exert an important role in the regulation of mitochondrial metabolism and composition under hypoxia, which has been discussed previously. HIF1α participates in glycolytic pathways and results in the conversion of energy production from OXPHOS to glycolysis. EVs and their active molecules can also target HIF1α to change cell metabolism. EVs derived from tumor-associated macrophages transport a myeloid-specific long noncoding RNA to enhance the aerobic glycolysis by binding PHD2 and preventing the degradation of HIF-1α, which promote the apoptotic resistance of breast cancer cells^110^. Ectosomal pyruvate kinase isoform M2 derived from hepatocellular carcinoma activates glycolysis via HIF-1α, and induces metabolic reprogramming in monocytes and macrophage differentiation^111^. HIF-1α inhibits mitochondrial oxidation by activating the PDK1, a HIF-1α target gene^112,113^. It is reported that EVs upregulates miR-302b in the receptor cells, evoking HIF1α/PDK1 pathway to affect glycolysis^114^. Thus, EVs might be a powerful tool for regulating mitochondrial energy metabolism.

#### PGC-1α pathway

Peroxisome proliferator-activated receptor γ (PPARγ) coactivator 1α (PGC-1α) is a 91 kDa nuclear factor that controls energy homeostasis such as OXPHOS, fatty acid oxidation, and mitochondrial biogenesis by interacting with estrogen-related receptors (ERRs), nuclear respiratory factors 1 and 2 (NRF-1/2), PPAR family of transcription factors to regulate mitochondrial genes^115,116^. Hypoxia can decrease the endothelial mitochondrial function through the negative influence on PGC-1α. Similarly, the increase of PGC-1α increased endothelial mitochondrial respiration, and improved cell function^117^, which may also be related to the metastasis of breast cancer^118^. Recently, it has been found that EVs can also affect phenotypic changes of receptor cells via targeting PGC-1α. For example, EVs secreted by perivascular adipose tissue are enriched in miR-221-3p, which can be delivered to vascular smooth muscle cells (VSMCs) to cause metabolic disorders and mitochondrial dysfunction, causing an early vascular remodeling by targeting PGC-1α^119^. Similarly, neuronal cells treated with adipose-derived stem cells-exosome showed increase of PGC-1α expression level, recovering mitochondrial function^120^. During inflammation, PGC-1α dysregulation induces the accumulation of ROS to modify the metabolic properties^121^, so PGC-1α may be an interesting therapeutic target.

#### Adenosine monophosphate‑activated protein kinase (AMPK) pathway

AMPK is not only the sensor and regulator that senses cellular energy^122^, but also regulates the mitochondrial biology and homeostasis, including mitochondrial biogenesis, mitophagy, and mitochondrial fission/fusion^29^. Hypoxia leads to mitochondrial damage and cell apoptosis, and also triggers the activation of AMPK to adapt to hypoxia^123^. AMPK regulates mitochondrial fission by increasing localization of DRP1 depending on the phosphorylation of mitochondrial fission factor (MFF)^124,125^, and activates autophagy-initiating kinase ULK1 directly to regulate mitophagy^126^. At sufficient energy levels, the activation of mTORC1 phosphorylated ULK1 to inhibit the AMPK-ULK1 interactions^127^.

A lncRNA upregulates SIRT1 by sponging miR-29a, promoting the recovery of mitochondrial function in cells of myocardial ischemia-reperfusion injury through activating AMPK/PGC1α signal pathway^128^. The exosomes secreted by MSCs overexpressed macrophage migration inhibitory factor inhibited mitochondrial fission by activating AMPK, thereby reducing the production of mROS and improving heart function in a rat with myocardial infarction^129^. Also, HIV-exosomes induce mitochondrial hyperfusion by reducing the phosphorylation of DRP1, and have an detrimental effect on brain endothelial function^130^. Therefore, finding a suitable target for the intervention of mitochondrial dynamics through EVs may have clinical therapeutic significance.

#### Fas/FasL pathway

Fas receptor is a member of the death receptor family, which exists on the surface of many cells and participates in cell death. Fas has beneficial and harmful effects on the immune cells by the interaction with its ligand FasL under different circumstance, and the pathological processes of cells can also be regulated^131^. ROS plays a key role in regulating cell function, and EVs can induce the production of mROS through Fas/FasL pathway, which leads to cellular dysfunction. EMPs secreted by apoptotic T cells activate neutral sphingomyelinase (SMase) by acting on Fas/FasL to increase cytoplasmic ROS, which activates endoplasmic reticulum (ER) stress, improving the production of mROS through the interaction between ER and mitochondria. When ER stress was inhibited, the production of mROS in endothelial cells treated with MPs decreased and the change of mitochondrial respiration was abolished, indicating that ER stress plays an important role in the production of mROS and the destruction of mitochondrial function, in which Fas receptor plays a key role in the alteration of mitochondrial function induced by EMPs^132^.

However, the understanding of the mechanism by which EVs regulate mitochondrial function is still limited. HIV-1-infected macrophage-derived exosomal-miR-27a altered the mitochondrial bioenergetics of recipient cells by targeting PPARγ^133^, and targeting miRNAs may improve lung health. Also, therapeutic targeting of mitochondrial reprogramming may represent strategies to disrupt oncogenic changes, and this reprogramming of mitochondrial dynamics can be induced by hypoxic EVs^134^.Thus, the combination of targeting specificity of EVs and their enriched cargo to affect the mitochondrial homeostasis of receptor cells can be expected to gain greater attraction in the future.

## Significance of mitochondria regulated by EVs in hypoxic diseases

### Significance for hypoxic/ischemic injury

Ischemia is a disruption of blood flow in tissue, resulting in the lack of oxygen. Ischemic injury includes traumatic hemorrhage, stoke, ischemic heart disease, acute kidney injury (AKI), organ transplantation, and others. The increase of mROS and mitochondrial fission are the important causes of hypoxic/ischemic injury, which can be regulated by EVs and their contents. For instance, plasma exosomes attenuate mitochondrial damage and inhibits the production of ROS by transferring HSP70 to the cerebral ischemia/reperfusion (I/R) injury area^135^. MSC-exosomes inhibits mitochondrial fission to improve heart function in myocardial infarction mouse model^129^. Similarly, MSC-derived EVs inhibit mitochondrial fission via the transfer of miR-30, which reduces apoptosis and alleviates renal I/R injury. These effects are reduced when miR-30 was inhibited, indicating that EVs-miRNA may be used as a diagnostic marker^136^. Also, the same effect was observed in exosomes carrying miR-210^137^. EVs-miRNA-200a-3p secreted by MSCs improve the mitochondrial function of tubular epithelia cells in renal I/R injury via acting on the Keap1-Nrf2 signaling pathway^138^. As the main organelle affected by ischemia/hypoxia injury, mitochondria can be used as the targets of EVs, so as to provide ideas in the diagnosis and treatment of diseases.

### Significance for hypoxic tumor diseases

Mitochondria play an important role in cancer through energy production, signal transmission and quality control. As early as 1956, Warburg suggested that cancers would maintain high glycolytic capacity in the presence of oxygen, which called aerobic glycolysis, and mitochondria were thought to be mere bystanders of cancer progression^139^, and then researchers gradually found out that mitochondria exert the vital effects on tumor metabolism, calcium homeostasis and control redox, oncogenic signaling, and cell death, besides supplying energy, which is closely associated with malignant tumor progression^2,38^. In addition, mitochondrial function can be regulated by hypoxia tumor exosomes. Park et al.^44^. found that exosomes-let-7a miRNA derived from hypoxic tumor cells enhanced OXPHOS in macrophages, favoring tumor development. Normal mammary epithelial cell treated with EVs derived from hypoxic human breast cancer cells activates Ser616 phosphorylation of DRP1 and increases the expression of MFN1 and MFN2 with mitochondria, and stimulates mitochondrial fission and fusion events, which is positively correlated with cell migration^134^. Also, Clement et al. demonstrated that increased gene expression of mitochondrial dynamics (FIS1, MFN2, and OPA1) correlated with decreased overall survival in melanoma patients, which can be provoked by the adipocytes via EVs, and promoting melanoma aggressiveness^109^. Resistant breast cancer cells derived EVs transported Hsp70 into mitochondria, enhancing adriamycin resistance^140^. Moreover, exosomes regulate multiple malignant changes by reprogramming of mitochondrial metabolism via shuttling metabolic related substances^108,109,111,141^, thus preventing EVs from transporting cargo might be an effective strategy for regulating mitochondria and cancer metastasis and progression.

## Prospects

In this review, we summarized the changes of mitochondria under hypoxia, and the role of mitochondria as signal organelles in regulating cellular phenotypic differentiation, inflammation and death. Mitochondria can maintain a stable state under hypoxia through mitochondrial quality control mechanism, such as mitochondrial metabolic homeostasis, mitophagy, mitochondrial fission and fusion, EVs, MDVs, as well as mitochondrial transfer via multiple pathways. Mitochondrial function could be regulated indirectly by EVs via the delivery of contents under hypoxia, facilitating tumor progression and ischemic damage. The delivery of mitochondria or mtDNA via EVs between cells under stress causes inflammatory response in the recipient cells. In addition, EVs derived from MSCs and some normal cells have a protective effect on mitochondria of recipient cells. Drugs have been used in tumors and metabolic diseases by regulating mitochondrial biosynthesis and metabolism, but there still are lack of clinical studies on EVs regulate mitochondrial function.

Although researchers have shown that EVs play an important role in the regulation of mitochondria, the specific substances contained in EV and whether these substances enter directly into the mitochondria of recipient cells are still unclear. Exosomes and microvesicles can be distinguished because of their different positions of origin and sizes, and their biogenetic mechanisms are similar but also different. And it is unclear whether their roles in the regulation of mitochondria are also different^76^. Better tracking models, monitoring methods and isolation techniques are the basis for further research. Anyway, this review will provide ideas for the research on the intervention of mitochondrial function through EVs.
